# Application of Genome-Wide Single Nucleotide Polymorphism Typing: Simple Association and Beyond

**DOI:** 10.1371/journal.pgen.0020150

**Published:** 2006-10-06

**Authors:** J. Raphael Gibbs, Andrew Singleton

**Affiliations:** University College London, United Kingdom

## Abstract

The International HapMap Project and the arrival of technologies that type more than 100,000 SNPs in a single experiment have made genome-wide single nucleotide polymorphism (GW-SNP) assay a realistic endeavor. This has sparked considerable debate regarding the promise of GW-SNP typing to identify genetic association in disease. As has already been shown, this approach has the potential to localize common genetic variation underlying disease risk. The data provided from this technology also lends itself to several other lines of investigation; autozygosity mapping in consanguineous families and outbred populations, direct detection of structural variation, admixture analysis, and other population genetic approaches. In this review we will discuss the potential uses and practical application of GW-SNP typing including those above and beyond simple association testing.

## Introduction

The first steps toward effective whole genome association experiments were taken with the inception and completion of stages I and II of the International HapMap Project (http://www.hapmap.org [1,2]). This project aimed to produce a minimal set of informative single nucleotide polymorphisms (SNPs) to tag variation throughout the genome [3]. In an effort to understand how the requirements for SNP tagging approaches may vary from population to population, HapMap data was initially generated in four discrete populations: Yoruba from Ibadan, Nigeria (YRI); Japanese in Tokyo, Japan (JPT); Han Chinese in Beijing, China (CHB); and Utah, United States, residents with ancestry from northern and western Europe (CEU).

A key effort undertaken in parallel with the HapMap Project involved the production of cost-effective methods to perform high-throughput genotyping accurately and reproducibly. There are two prominent companies offering high-throughput genome-wide (GW) genotyping that can be applied within an end user's laboratory: Affymetrix and Illumina. The combination of these technological and informatic advances now make GW-SNP genotyping a realistic possibility for well-funded laboratories; the likely decrease in cost that will occur over the next five years suggests that this technology will become a standard technique in molecular genetic and clinical diagnostic laboratories. In this review article we will discuss the potential applications and practical considerations of GW-SNP assay. While we have experience in dealing with large datasets (~.5 billion genotypes) from both Affymetrix and Illumina technologies, much of this article focuses on the output and metrics produced using the Illumina Infinium assays, because our primary in-house work has centered on this platform. However, many of the concepts and applications discussed here are applicable to data derived from other platforms.

## Genome-Wide Association

GW-SNP assays have been anticipated as a tool for the dissection of disease risk factors for many years [4]. Much of the discussion surrounding the application of GW-SNP assays has centered on the utility of this method in identifying common genetic variability that underlies disease [5,6]. This discussion has focused on the relative power of these types of study and the potential problems and pitfalls associated with this approach, resulting in numerous review and opinion pieces. For the sake of brevity we will not discuss these considerations in detail. Briefly, however, the primary concern before undertaking a genome-wide association experiment is one of statistical power to observe an effect of a specific size. To date this issue has largely been addressed by prospective power calculations using simulation. These analyses generally rely on parameters such as the model of disease risk (dominant, recessive, additive) and estimates of the presence and magnitude of genetic and allelic heterogeneity; in reality the extent of genetic influence and genetic mode of action for individual loci within most diseases is unknown, and most of these approaches do not consider the confound of population stratification [7,8], thus these predictions are essentially “best-guess” estimates.

Highlighting the approximate nature of these calculations, the arbitrary number of 1,000 cases and 1,000 matched controls appears to have been adopted as the realistic standard for complex diseases. Compared with most genetic case control studies, which typically number a few hundred cases and controls, 1,000 samples in each cohort is relatively large; however, it is doubtful that even sample series of this size will provide sufficient power to identify recessive loci and less likely that the identification of gene–gene or gene–environment interactions will be tenable. Nevertheless, this size of study appears to be an achievable goal, although currently only for consortia or particularly well-funded laboratories. The considerable cost of these experiments coupled with the potential promise of this approach has led funding agencies to encourage sharing of resources to perform these assays, including both sharing of DNA samples and public release of genotype data. Implicitly, this policy highlights a strength of GW-SNP experiments, i.e., genotype data are essentially digital and additive; thus experiments on the same platform can be easily compared or combined to increase power and sensitivity. The public release of genotype data inevitably raises issues with respect to patient privacy and appropriate clinical research protocols and consenting procedures. While these restrictions may preclude use of some existing collections for publicly funded experiments, with adequate foresight these issues are not insurmountable. Indeed initiatives such as the Genetic Association Information Network (http://www.fnih.org/GAIN/GAIN_home.shtml) and the Wellcome Trust Case Control Consortium (http://www.wtccc.org.uk) will release genotype data, albeit with varying restrictions on who can access the data.

Another consideration in the design of GW-SNP experiments is the choice of source tissue; although the amount of DNA required is relatively small, the experiments are exquisitely sensitive to DNA quality. Consequently, quality of the sample source material may be as important as concerns over study design and sample size. Many large-scale experiments, particularly those publicly funded, will be performed on samples from public open-access repositories, indeed these resources were set up in anticipation of GW-SNP and other high-throughput methodologies. Repositories have commonly used Epstein-Barr virus immortalization to create lymphoblast cell lines (LCLs) primarily to provide a renewable source of high-quality genetic material. An understandable concern is the fidelity of the genomic DNA in these cultured cells when compared with that from the source tissue. Experiments within our laboratory suggest that genomic DNA from LCLs produces GW-SNP data that is relatively faithful to that derived from the blood samples used to create the LCLs; the LCL creation and passage process results in alterations in less than 0.1% genotype calls when compared directly with the source tissue. In terms of genome-wide association, such a small and apparently randomly distributed alteration is unlikely to confound results. An additional and increasingly popular method for generating large amounts of genomic DNA is genome-wide amplification (GWA). This technique allows the production of significant amounts of genomic DNA from a variety of sources, including archived fixed tissue. This approach is of obvious utility, particularly where samples are rare or are individual fixed samples of particular importance. There is currently limited data on the use of this technology in the creation of starting material for GW-SNP assay; however, experiments assessing this approach in high-plex assays of >2,000 SNPs show a high degree of genotype concordance between amplified and source material [9]. While these data suggest that GWA will be a viable approach to generate material for GW-SNP assay, it is clear that the initial template must be good quality high molecular weight DNA; the potential for failed genotypes or allelic bias as a result of amplification artifacts or contamination is likely to be inversely related to sample quality.

Although GW-SNP assays have only been available for a short period, several studies using this approach have already been published [10–12]. One such study resulted in the discovery of variability within *CFH* (complement factor H) as a strong risk factor for age-related macular degeneration [11]. This work used a relatively small number of SNPs (approximately 100,000) in a small cohort of 96 cases and 50 controls. The primary data showed strong association at two SNPs within *CFH;* resequencing revealed the presence of a coding alteration in *CFH* associated with a 7-fold increase in risk of disease for homozygous carriers. Not only did this study highlight the utility of GW-SNP assay but it also showed that relatively small case-control series can provide valuable information.

Perhaps as important as defining a positive signal, GW-SNP association studies may also define the limits of influence of common genetic variability in a particular disorder. One can, in the absence of the requisite significant signal, show that there is no common variant that confers a risk stronger than a predetermined odds ratio in the population studied. Put simply, in a perfectly designed experiment this approach has the power not only to tell you what is there, but also to tell you what is not there. Of course in the real world where likely confounds such as population stratification and incomplete genomic coverage [13,14] lead to suboptimal experimental design, the absence of evidence is not necessarily evidence of absence, and of course the results from a single experiment are only directly relevant to the population studied. However, genomic coverage is increasing, genomic control or principal component analysis [15] may reduce stratification, and our understanding of interpopulation genetic heterogeneity over the next decade is likely to improve. Thus while these experiments are unlikely to unequivocally rule out the presence of other simple genetic influences in a particular trait, they do provide a reasonable reference point with which we can make estimates of the presence/absence of loci. This has particular relevance for funding agencies and scientists alike; it tells us when we should stop looking for a common genetic variant at a certain power.

## Considerations for Data Handling and Analysis

As the density of GW-SNP genotyping platforms continues to increase, it is plausible that many laboratories will attempt to manage billions of genotypes per year. Whether laboratories are producing these genotypes directly, through collaborations, or retrieving them from a public resource (http://www.ncbi.nlm.nih.gov/WGA), the challenge of data volume still remains.

An obvious choice for data management is to use a relational database, which offers many levels of storage (including backup) and access (with privileges) to the data. Storing the data in a database makes the task of generally mundane but important jobs fairly easy, and facilitates the ability to use the data in many different forms, such as exporting for multiple analysis formats. An issue specific to the storage of SNP genotypes, which may affect which encoding is used to store a genotype and its sample and marker relationship, is that they have a direction and orientation; this latter point is of particular importance as even within platforms a SNP's encoded orientation may change between product releases. Because of the storage requirements (each chip image may be 2 Gb in size), typically chip images and other chip artifacts will be archived with a low level of access or discarded once genotypes have been called; however, as noted below many researchers will have interest in data above and beyond genotypes, wishing to access metrics that provide information on copy number. Thus investigators should expect to store relevant metrics in addition to the raw genotypes.

Available tools for data management and analysis of GW-SNP data are currently scarce; however, a few exist or are under development, such as GERON Genotyping (http://neurogenetics.nia.nih.gov), Snpgwa (personal communication Drs. Langfeld and Steigert, Wake Forest University), and PLINK (http://pngu.mgh.harvard.edu/~purcell/plink). These new tools should perform whole genome association analysis quickly; especially when compute clusters are available for parallel execution. Analyses within Snpgwa and PLINK include tests for allelic, genotypic, dominant, recessive, and additive model associations as well as tests for linkage disequilibrium and Hardy-Weinberg equilibrium. These tools also allow testing of haplotype association in consecutive (Snpgwa) or selected (PLINK) markers. Association analysis with permutations and multiple testing corrections are available, but these steps may add considerably to computational time. An underestimated and as yet unsolved problem with GW-SNP data is that of data visualization, particularly integration of results with current genomic data.

Some of these tools will be made available under an open-source license, which is an important model to consider. In much the same way that sharing the resulting GW-SNP data should have an additive effect for the advancement of biological understanding, this should apply to the software tools as well. While sharing these toolsets alone may be sufficient for repeatable analysis and data handling, doing so under on open-source license has additional benefits. The open-source model lends itself well to the formation of a collaborative community in the development, extension, and use of software; additionally this adds to the diversity of functionality, speed with which changes may be made, and scrutiny of the correctness and efficiency of implemented algorithms.

## Homozygosity Mapping in Disease

Perhaps the most obvious application of this technology outside of allelic, haplotypic, and genotypic association testing is in the detection of tracts of extended homozygosity, particularly those believed to contain a genetic lesion underlying disease. While the concept of autozygosity mapping was first proposed in 1987 [16], the advent of GW-SNP assay offers many advantages over previous methods aimed to define recessive loci. In kindred with an apparently recessive disorder, particularly one where parental consanguinity is suspected, this approach can be used to map regions of extended homozygosity with high resolution and essentially complete genomic coverage. These assays can be completed in a short amount of time; typically GW-SNP assay of a family of fewer than 50 individuals takes less than a week. The data lends itself to immediate visualization of large homozygous tracts (Figure 1). Because all tracts of disease segregating homozygosity will be identified and all heterozygous regions/nonsegregating homozygous tracts excluded, one can be confident that the region harboring the genetic lesion underlying disease has been identified; thus, with the caveat that the model needs to be correct (i.e., the disease must be caused by a homozygous change inherited from a relatively recent common ancestor), generation of lod scores for these types of analysis is effectively redundant.

**Figure 1:**
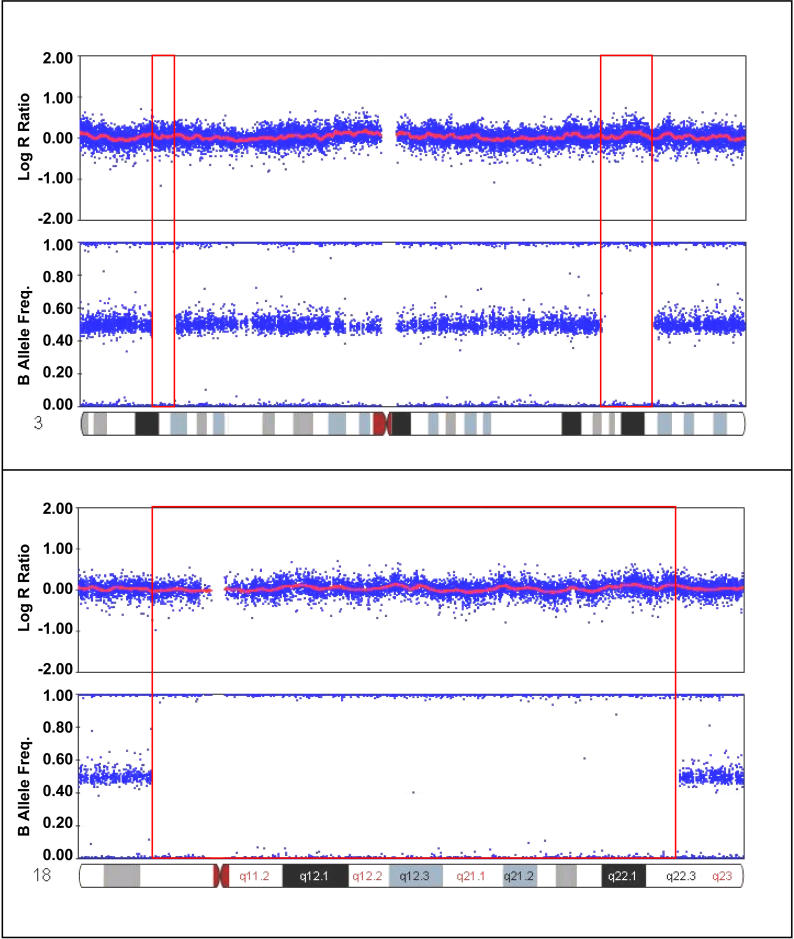
Multiple Regions of Extended Homozygosity within a Single Subject Born from a First Cousin Marriage The regions of extended homozygosity on Chromosomes 3 (upper panel) and 18 (lower panel) are outlined in red. Homozygosity is shown by a lack of A/B genotype calls (corresponding to a B allele frequency of 0.5) coupled with a normal copy number, indicated by an average log R ratio of 0.

Whether this technique will identify single or multiple regions of interest and the size of these regions relies on several factors; the degree of parental consanguinity, the number of informative family members, and the relatively stochastic nature of recombination. In small families where there are fewer meioses and thus more chance for variation in the length and number of homozygous tracts, power calculation for these types of experiments will generally be uninformative.

In families where affected family members exhibit a low level of inbreeding, or where there is a high degree of separation between affected family members, the size of a potential disease-segregating region is likely to be small. In this event the high level of resolution provided by GW-SNP assay offers a great advantage over traditional genome-wide linkage scans.

The family-based work logically leads to another application, that this methodology may be used to perform autozygosity mapping for disease in populations of individuals with unknown pedigree structures [17]. This suggests the possibility of performing autozygosity mapping in cohorts ranging from relatively conserved populations to those believed to be ostensibly outbred. Data from our laboratory has shown a surprisingly high level of apparent parental consanguinity in aged North Americans, with >6% of sampled individuals possessing tracts of homozygous genotypes larger than 5 Mb (unpublished data), and these observations are consistent with analyses of HapMap data [18]. One could expect therefore that this methodology may be applied to localize homozygous genetic mutations underlying disease. In addition to speed and resolution, GW-SNP assay offers one more advantage in autozygosity mapping: this technique allows the direct visualization of structural genetic mutation such as genomic deletion and duplication, which often underlie recessive disorders.

## Direct Detection of Structural Variability

Two primary metrics produced by the Illumina Infinium technology allow the direct visualization of structural genomic variability, B allele frequency, and normalized R. Because there is considerable redundancy at each locus interrogated, both alleles of each SNP are assayed multiple times; accordingly, B allele value for an individual SNP in a single sample gives an estimate of the proportion of times an individual allele at each polymorphism was called A or B. This metric is simply viewed as B allele frequency, thus an individual homozygous for the B allele would have a score close to 1, an individual homozygous for the A allele a score close to 0, and a score of approximately 0.5 would indicate a heterozygous A/B genotype. Deviations away from these three cluster positions are indicative of a deviation from a simple diplotype. R is a measure of the signal intensity for a locus, and thus when compared with an average expected value for that locus, the resulting log R ratio provides an indirect measure of copy number. A signal in the test sample which is stronger than the expected signal is indicated by a log R ratio above one and indicates a copy number increase; conversely a weaker signal than that expected for an individual SNP results in a decreased log R ratio and is suggestive of a deletion.

Visualization of B allele frequency and log R ratio can be used to identify genomic copy number variation in individual samples (Figure 2). Currently, analysis of these data needs to be performed manually. This coupled with the fact that there is a small amount of interassay variation in the metrics B allele frequency and R at individual loci means that identifying copy number variations affecting a single SNP or a small number of SNPs is challenging; however, copy number variation affecting ten or more contiguous SNPs is relatively easy to detect. Thus the resolution of this technique is sensitive to SNP density of the assay at any particular genomic locus. For all platforms this varies throughout the genome. However, typically we observe copy number variations as small as 200 kb using the Infinium I assays (109,365 SNPs) and as small as 50 kb using the Infinium II assays (317,511 SNPs).

**Figure 2:**
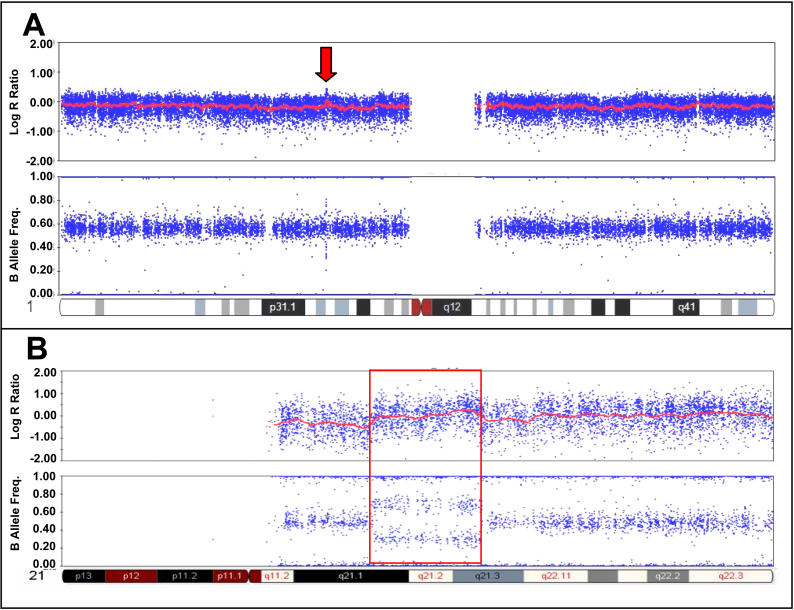
Log R Ratio and B Allele plots for Two Samples Showing Genomic Duplication Genomic duplication is indicated by an increase in log R ratio and B allele frequency clusters outside of the expected values of 1 (B/B), 0.5 (A/B), and 0 (A/A). (A) Shows duplication of a small segment on Chromosome 1 (red arrow). (B) Shows duplication of a region of approximately 7 Mb on Chromosome 21, outlined in red. This region contains APP, and the duplication mutation results in a neurodegenerative phenotype (courtesy of J. Hardy).

The ability to view and score structural genomic variation has many potential applications. Most obviously it allows the analysis of copy number polymorphisms as disease risk alleles in GW-SNP association experiments. Given the biological plausibility of a role for altered gene dose/expression in disease, this is of particular interest. This assay also lends itself to the detection of structural mutation underlying monogenic disease; within our laboratory we have detected deletion and duplication of *PRKN* in addition to multiplication mutations across *SNCA* and *APP* (unpublished data; Figure 2).

This assay may also be used to assess the role and extent of somatic structural mutation, an application that is immediately applicable to cancer but conceivably may be used to assess this phenomenon in other disorders. While analysis of copy number variation has been performed previously using custom clone or oligonucleotide arrays for comparative genomic hybridization, these products have often provided limited coverage in a format that is either not reproducible or not easily transferable between laboratories. The availability of a transferable technology that can provide similar results in a highly reproducible manner is a significant step toward providing a standardized encyclopedia of both somatic and germ line structural genomic variation and thus aid in defining the relationship of this variation to traits such as disease.

## Other Applications of GW-SNP Assay

It is worth briefly noting a few potential applications of GW-SNP assay beyond those described above.

The application of this technique for detection of structural mutation suggests its use in the genomic characterization of laboratory resources. This could include analysis of human-derived cell lines that are currently used on a daily basis within laboratories around the world in addition to the characterization and monitoring of newly created lines such as stem cells. Data from our laboratory and others shows both genomic instability in these lines and the utility of GW-SNP typing in the molecular characterization of cell lines [19] (Figure 3); routine monitoring of the genomic variability within lines will add another layer of quality control and facilitate more direct comparison between laboratories working on lines derived from a common founder. With the release of similar products for other species, it is also expected that these assays will greatly facilitate the analysis of other laboratory resources; for example GW-SNP assay would quickly reveal the extent to which a particular mouse line was congenic and also allow rapid targeted breeding of animals.

**Figure 3:**
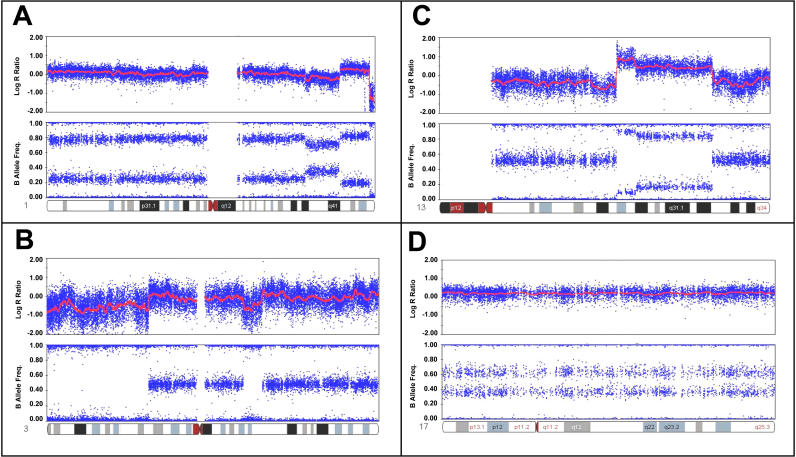
GW-SNP Assay Using the Illumina Infinium II Arrays Reveals Structural Variation in Chromosomes (A) Common laboratory cell line HEK293. (B) Common laboratory cell line M17. (C) Human embryonic stem cell line 293F7. (D) Human embryonic stem cell line Bg01P3SFF2. These include apparent multiplication and deletion mutations in the M17 and HEK293 lines, multiplication mutations across line 293F7, and duplication Chromosome 17 in line Bg01P3SFF2.

In terms of population-based applications, the density of these assays and the low mutation rate of SNPs suggest that these assays will lend themselves to the genetic characterization of individuals based on genetic background. The most comprehensive attempt at performing this type of analysis was previously performed using microsatellite markers in a panel of approximately 1,064 individuals from 51 distinct populations [20]. While this effort was successful in anonymously dividing the subjects into their correct geographic origins, the production of these data was an enormous undertaking, and it would be difficult to apply these experiments to a single sample. Some attempt at defining population-specific alleles has been performed in the typing of YRI, CHB, CEU, and JPT subjects; however, GW-SNP assay of a broader array of populations promises not only to provide information for admixture analyses but also insight into genetic drift, relative inbreeding, and genetic selection across and within populations [3,17].

## Conclusions

The field of research surrounding GW-SNP assay has taken enormous leaps forward in the last five years. The International HapMap Project has helped to define informative genetic variation, and reliable, reproducible technology capable of leveraging this information is now realistically available.

In the next period of development it is likely that the increasing SNP density will begin to level out as the cost/benefit ratio of increased information content decreases. This will result in a product that has sufficient coverage to assay most populations or one that can easily be augmented with population-specific SNPs to increase coverage in a particular population of interest.

The advantages of this method are apparent; it offers unparalleled density and provides accurate data quickly. The digital nature of these data means that it is easily transferable and experiments can be performed in an additive nature across laboratories. The promise of this method in revealing the role of common genetic variability in disease is beginning to be realized, and it is clear that there are many applications of these data above and beyond simple genetic association.

## Supporting Information

### Accession Numbers

The accession numbers from the Entrez Gene databank (http://www.ncbi.nlm.nih.gov/entrez/query.fcgi?db=gene) are for: multiplication mutations across *APP* (351); *CFH,* complement factor H (3075), *PRKN* (5071); and multiplication mutations across *SNCA* (6622).

## Abbreviations

GW-SNP: genome-wide single nucleotide polymorphism
GWA: genome-wide amplification
LCLs: lymphoblast cell lines
